# Prevalence of human papillomavirus detection in ovarian cancer: a meta-analysis

**DOI:** 10.1007/s10096-021-04282-7

**Published:** 2021-06-04

**Authors:** Soumia Cherif, Abdessamad Amine, Sarah Thies, Eliane T. Taube, Elena Ioana Braicu, Jalid Sehouli, Andreas M. Kaufmann

**Affiliations:** 1 Charité – Universitätsmedizin Berlin, corporate member of Freie Universität Berlin and Humboldt-Universität zu Berlin, Department of Gynecology, Augustenburger Platz 1, 13353 Berlin, Germany; 2 Laboratory of Biochemistry, Environment, and Agrifood, Faculty of Sciences and Techniques-Mohammedia, Hassan II University, 8 Casablanca, Morocco; 3 Charité – Universitätsmedizin Berlin, corporate member of Freie Universität Berlin and Humboldt-Universität zu Berlin, Institute for Pathology, Charitéplatz 1, 10117 Berlin, Germany

**Keywords:** HPV prevalence, Ovarian malignancy, Worldwide, Subgroup analysis, Etiologic agent

## Abstract

We conducted a meta-analysis of published data to update and estimate the prevalence of HPV in ovarian cancer. A comprehensive literature search was performed according to the PRISMA guidelines. Eligible articles published from 1989 until 2020 by searching Web of Sciences, Pubmed, Embase, and the Cochrane Library Central databases were gathered. A pooled estimation of HPV prevalence with a 95% confidence interval (CI) was calculated based on a random effect model. Quantitative assessment of heterogeneity was explored using Cochrane test and *I*^2^. Additionally, publication bias, sensitivity, meta-regression, and subgroup analyses were also performed. Twenty-nine studies involving 2280 patients with ovarian cancer were included. The statistical heterogeneity was high (*I*^2^ = 88%, *P*<0.0001). The pooled prevalence of HPV in ovarian cancer cases was 15.9% (95% CI, 11–22). In subgroup analyses, the highest prevalence of HPV was reported by studies from Asia (30.9%; 95% CI, 20–44) and Eastern Europe (29.3%; 95% CI, 4.4–78). Furthermore, the most frequently detected HPV genotype was HPV16 (54%; 95% CI, 27.9–55), followed by HPV18 (23.2%; 95% CI, 18.8–28.2). Our meta-analysis suggests a great difference in the prevalence of HPV detected in ovarian cancer by different studies, which is not seen in strongly HPV-associated cancers such as cervical cancer. However, the prevalence varied markedly by geographic region. Considering the substantial heterogeneity found, more studies with control groups and precise assays measuring HPV mRNA expression are needed to further evaluate the link and causative aetiology between HPV and ovarian cancer.

**Supplementary Information:**

The online version contains supplementary material available at 10.1007/s10096-021-04282-7.

## Background

Ovarian cancer remains the most life-threatening malignancy of the female genital tract mainly because of the lack of early clinical symptoms and early detection (FIGO stage I–II) [1]. The stage of ovarian cancer is an important prognostic factor at diagnosis [2, 3]. This malignancy is typically diagnosed at a late stage (FIGO stage III–IV) with a 5 year-survival rate reaching 20% whereas it is 80–90% for patients with localized tumor (stage I) [4, 5]. Despite the considerable advances in highlighting risk factors, the pathogenesis and aetiology of ovarian cancer are still unclear [1]. However, an increased risk of developing ovarian cancer has been strongly correlated to genetic mutations (BRCA1 or BRCA2 genes), family history of ovarian, breast, or colon cancer, age, postmenopausal hormonal therapy use, infertility, and nulliparity [4]. Besides these findings, a current theory, supported by epidemiological data, hypothesizes that persistent viral infection and chronic inflammation may play a role in the carcinogenesis of ovarian cancer [2]. Thus, it is hypothesized that viral infection may contribute to ovarian cancer.

HPV has been identified as an etiological agent of numerous proliferative epithelial lesions in the skin and various mucosal sites, including the lower genital tract (of both sexes) and diverse sites of the oropharyngeal area and the upper aerodigestive tract [6]. However, its role in the development of cancers in the upper genital tract, such as endometrial and ovarian cancer is still under debate [7]. In fact, studies carried out so far failed to associate firmly the presence of HPV with the occurrence of these malignancies. Some reported a positive correlation while others indicated negative results [1, 6, 8]. These discordant findings might be due to the fact that most studies on this issue were observational reports without including control groups [2, 9]. Furthermore, controversial results might be due to variation in the geographic distribution of HPV, and the technique used to identify HPV infection.

Two systematic reviews and meta-analyses have studied the association between HPV and ovarian cancer by investigating the prevalence of HPV in ovarian cancer tissues [2, 10]. The results, however, were conflicting. Besides, data from the Middle East had not been included. As more studies concerning HPV detection in ovarian cancer have been published recently, we undertook the present meta-analysis to update and better define this relationship.

## Material and methods

### Study identification and data extraction

Two investigators (S.C. and A.A.) performed a systematic literature research independently by using Pubmed, Embase, and Cochrane Library Central between 1989 until 2020, using the following Medical Subject Heading terms (MeSH) and text words: “Human papillomavirus”, “HPV”, “ovarian malignancies”, “ovarian neoplasm”, and “ovarian cancer”. The investigators extracted independently data from identified studies; additional studies were retrieved and reviewed. In the case of discrepancy, the decision on inclusion/exclusion was made by discussion. The meta-analysis was performed in agreement with PRISMA criteria.

Data extraction was carried out, in an Excel sheet, to record details of the first author, country of publication, method of detection, histological type, type of specimen, sample size, HPV genotype, and numbers of HPV-positive and negative patients.

### Study selection

The following criteria had to be met to include studies in this meta-analysis: (1) observational studies published between 1989 until 2020 with data on the association of HPV in ovarian cancer and including at least 5 cases, (2) written in English, and (3) published as a full peer-reviewed article. The exclusion criteria were the following: (1) studies where a serological test is used to detect HPV, (2) studies not meeting the inclusion criteria, and (3) studies limited to animals.

### Statistical analysis

The pooled prevalence of HPV in ovarian cancer and 95% confidence interval (CI) were calculated by using the Mantel-Haenszel method of DerSimonian and Laird method (random-effects or fixed-effects model). The heterogeneity was measured by the Cochran *Q* test (*P* < 0.10 demonstrates a high level of heterogeneity) [11, 12]. Moreover, the rate of inconsistency (*I*^2^) was also calculated (values of *I*^2^ from 50 to 75% correspond to moderate to high degrees of heterogeneity, respectively) [13]. If the heterogeneity was not substantial, the pooled rate with a 95% confidence interval (95%CI) was calculated using the fixed-effect model. When statistically significant heterogeneity was found (*I*^2^ >50), the pooled rate with 95% CI was calculated based on the random-effects model. Then, a subgroup analysis was employed to evaluate the influence of several factors on the overall results. A sensitivity analysis was also performed, by excluding one study each time, to assess the impact of each study on the strength and stability of our results. Meta-regression analysis was used to examine the association of the geographical distribution of the studies, specimen type and detection methods with the prevalence of HPV. The statistical analyses were performed by using the Comprehensive Meta-analysis software, version 3 (Englewood, USA).

### Publication bias

The publication bias was evaluated visually by constructing a Funnel plot. It was created by plotting the log prevalence of HPV vs the standard error. Their symmetry was evaluated by Egger’s regression test and the Begg and Mazumdar adjusted rank correlation test, all *P*-values were set on two sides, those less than 0.05 were deemed statistically significant.

## Results

### Description of studies

**Fig. 1:**
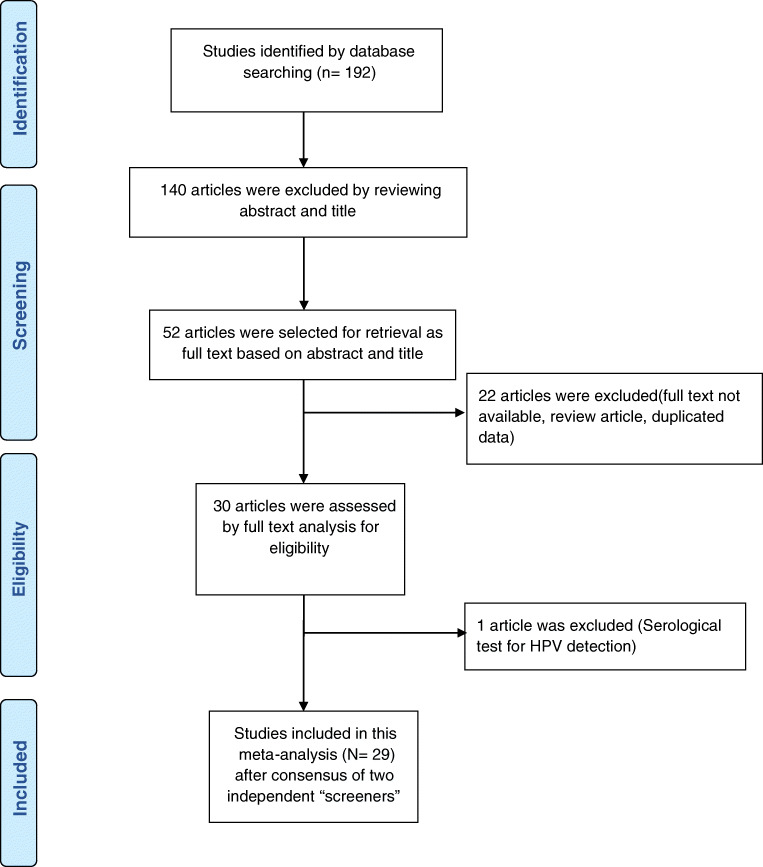
Flow diagram of the studies selected for this meta-analysis

Of the 192 articles initially identified by the investigators, 29 studies were included in this meta-analysis. The flowchart explaining the study selection is displayed in Fig. 1. The main characteristics of the studies are presented in Supplementary information 1. In 19/29 studies, the histological types were mentioned.

A total of 2280 patients were included in this meta-analysis. There were 6 studies from North America [14–19], 8 studies from the Middle East [1, 8, 20–25], 7 studies were conducted in Asia [7, 26–31] and the remaining 8 studies were reported from Europe [6, 9, 32–37]. As for the type of specimen utilized, Formalin-Fixed Paraffin-Embedded (FPPE) tissues were used in 19 studies [1, 6, 8, 9, 15, 17, 18, 20–27, 29, 30, 33, 36], in the remaining studies, frozen [7, 14, 19, 31, 32, 34, 35, 37] and fresh tissues [16, 28] were utilized.

The most used technique for the detection of HPV was PCR (25 studies) using the L1 consensus primers either by a single-step PCR with GP5+/GP6+ [9, 25, 30, 32, 34, 36] or MY09/MY11 [16, 17, 20] primer set or by nested PCR using primer pairs GP5+/GP6 + and MY09/MY11 [1, 8, 21, 22]. In 7 studies where specific primers for high-risk HPV and low-risk HPV [6, 7, 14, 15, 18, 29, 37] were used, 2 studies used commercial PCR kits (High-risk Human Papillomavirus Multiplex screening genesig® kit and HPV detection Madison® kit) [14, 37], whereas others used specific primers for E6/E7 regions [6, 7, 15, 18, 29]. Also, specific primers for HPV16 and 18 were utilized in one study [7]. Besides, four studies combined detection techniques either by using PCR with immunohistochemistry (IHC) or in situ hybridization (ISH) [19, 33] or by combining ISH with IHC [24, 27].

Generally, HPV16 [1, 7, 21–23, 25, 27, 28, 32, 33, 37] and HPV18 [1, 6, 7, 21, 26, 28, 33] were the most common genotypes detected. Also, cases with HPV33 [1, 20, 26], HPV45 [21, 22], and HPV6 [20, 31] were found. Multiple infections were also reported, in 3 studies a coinfection with two HPV types was reported [21, 22, 29]. Furthermore, in 2 studies, a coinfection with three HPV types was detected [21, 22] (Table 1).

**Table 1:** Subgroup characteristics of HPV in ovarian cancer and borderline lesions by region, HPV genotypes, detection methods, and biological specimens of the included studies

Subgroup	No. of studies	HPV+/total cases	Range of HPV positivity	Prevalence % (95% CI)
Total	29	428/2280	0–81%	15.9 (11–22)
Geographic distribution				
Asia	7	215/839	6–70%	30 (20–44)
Eastern Europe	3	32/134	7.4–81.5%	29.3 (4.4–78)
Middle East	8	175/665	1.9–42%	21.6 (13.2–33.4)
Southern Europe	1	3/71	-	4.225 (1.151–11.702)
Western Europe	4	1/442	0–0.7%	0.226 (0.011–1.270)
North America	6	0/129	-	0 (0–0.02)
Specimen				
FFPE	19	359/1848	0.5%–52%	16.7 (11.6–23.4)
Frozen tissues	8	66/348	0.7–81%	14.1 (3–40)
Fresh tissues	2	4/74	2–6.7%	5.8 (2.3–13.8)
Detection method				
Combined techniques (PCR/IHC; PCR/Southern hybridization; IHC/ISH)	4	50/161	2.6–52%	31.06 (24.4–38.5)
Nested-PCR (L1 Consensus primers GP5+/GP6+ and MY09/MY11)	4	124/426	1.9–42%	29.1 (25–33)
PCR (L1 consensus primer and specific HPV-type primers)	4	91/486	0.7–70%	18.7 (15.5–22.4)
Single-step PCR (L1 Consensus primers GP5+/GP6+ or MY09/MY11)	9	118/819	0.5–35.7%	14.4 (12–17)
PCR (specific primers for HR-HPV and LR-HPV)	7	45/353	0.5–81.5%	12.7 (4.7–10.3)
IHC	1	3/35	-	8.5 (2.9–22.3)
HPV genotypes				
HPV16	15	145/266	0–100%	54 (27.9–55)
HPV18	14	72/304	0–100%	23.6 (18.8–28.26)
HPV33	8	42/346	0–70%	17 (10.2–18.06)
HPV45	7	7/138	0–7.1%	3.8 (0.8–4.3)
HPV6	8	30/246	0–100%	12 (6.9–13.5)
HPV16/18	3	20/116	15.2–100%	17.2 (4.4–20.3)
HPV16/45	2	11/114	2.3–13.8%	9.6 (1.9–10.2)
HPV16/18/45	2	4/114	2.3–4.1%	3.5 (0.3–4.5)
HPV18/45	2	3/114	2.3–2.7%	2.6 (0.2–3.9)

*PCR*, polymerase chain reaction; *HPV*, human papillomavirus; *FFPE*, Formalin-Fixed Paraffin-Embedded; 506 *ISH*: In-situ hybridization

### Meta-analysis

In our Meta-analysis, significant heterogeneity between the different included studies was observed: *Q*= 231.4, df(*Q*)= 28, *I*^2^=88%, *P*≤0.001. *Q* represents the distance of each study from the mean effect, if studies have the same rate of prevalence, *Q* would be equal to df (degree of freedom). In our study, *Q* is higher than df, which is an evidence of variation between studies.

**Fig. 2:**
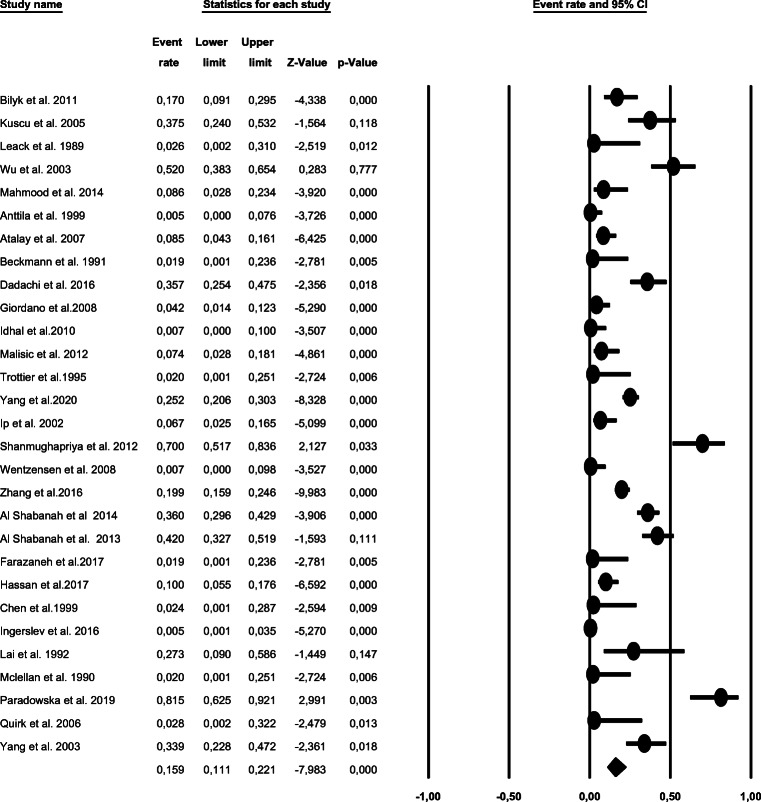
Forest plot showing HPV rate and 95% confidence intervals in ovarian cancer studies based on random-effect model

Because of the substantial heterogeneity found (*I*^2^= 88%), we applied the random-effect Model, with a pooled rate of 15.9% (95% CI, 11–22). Besides, a large variation of HPV prevalence across the included studies was observed (range 0–81%). The test for the overall effect is Z= −7.5, *P* ≤0.001 (Fig. 2). Thus, the null hypothesis was rejected that the true prevalence of HPV is comparable between the included studies.

### Subgroup and meta-regression analyses

A subgroup analysis was performed to investigate if the results found for all studies included would apply separately for the stratified data according to geographical region, type of specimen, or detection method. The results of the subgroup analysis are shown in Tables 1 and 2.

**Table 2:** Subgroup analysis by categorizing the data according to the geographic region, HPV genotypes, detection methods, and biological specimens of the included studies

Subgroups	Test of null (2-tail)		Heterogeneity		
	*Z* value	*P*-value	*Q*-value	df(*Q*)	*P*-value
Geographical region					
Asia	−2.7	0.006			
Eastern Europe	−0.7	0.4			
Middle East	−4.2	<0.001			
North America	−6.460	<0.001			
Southern Europe	−5290	<0.001			
Western Europe	−8145	<0.001			
Overall	−10.123	<0.001	59.4	5	<0.001
Specimen					
FFPE	−7.4	<0.001			
Fresh Tissues	−5.7	<0.001			
Frozen tissues	−2.5	0.01			
Overall	−9.1	<0.001	4.9	2	0.08
Detection method					
PCR (L1 Consensus primers GP5+/GP6+ and MY09/MY11)	−2.7	0.007			
PCR (L1 Consensus primers GP5+/GP6+ or MY09/MY11)	−5.9	<0.001			
PCR (specific primers for HR-HPV and LR-HPV)	−2.5	0.01			
PCR (L1 consensus primer and specific HPV-type primers)	−9.9	0.04			
Combined techniques (PCR/IHC; PCR/Southern hybridization; IHC/ISH)	−1.8	0.06			
IHC	−3.92	<0.001			
Overall	−7.9	<0.001	31.8	5	0.1

*PCR*, polymerase chain reaction; *HPV*, human papillomavirus; *FFPE*, Formalin-Fixed Paraffin-Embedded; 506 *ISH*: In-situ hybridization

Stratifying by geographical region, the highest rates of HPV detection in ovarian cancer tissue were found in Asia (30.9%; 95% CI, 20–44) ranging from 6% to 70%, Eastern Europe (29.3%; 95% CI, 4.4–78; range, 7.4–81.5%), and in the Middle East (21.6 %; 95% CI, 13–33.4; range, 1.9–42%). The lowest prevalence was detected in North America (0%; 95% CI, 0–0.02) (Table 1). Also, the test of the null hypothesis yields a *Q*-value =59.4, df(*Q*)=5, and *P*-value <0.001 (Table 2), indicating that the effect size differs by geographical regions. Of note also within a given region the variation in prevalence is substantial.

Results of subgroup analysis based on specimen types suggest that HPV rate is slightly greater when FFPE tissues were examined (16.7%; 95% CI, 11.6–23.4) compared to frozen tissue (14.1%; 95% CI, 3–39.9) or fresh tissue (5.8%; 95% CI, 2.3–13.8). The overall *Q*-value, df(*Q*) and *P*-value for specimen type subgroup were 4.9 with 2 df and 0.08, respectively. Thus, statistically, the effect size does not differ by the specimen type.

Regarding detection methods of HPV, the lowest prevalence of HPV is found when IHC is used (8.5%; 95% CI, 2.9–22.3) [23]. A greater prevalence was observed when more sensitive or combined techniques (PCR and ISH or IHC; southern hybridization and IHC) (31%; 95%CI, 24.4–38.5) were utilized. For the detection method stratification, the overall *Q*-value is 31 with 5 df, and the corresponding *P*-value is 0.1. Thus, the effect size does not differ significantly by the detection method.

Taken together, the meta-regression analyses showed a significant association of HPV prevalence with geographic distribution (*P*≤ 0.001). However, the specimen type (*P*=0.19) and HPV detection methods (*P*=0.3) were not statistically associated with HPV prevalence.

### Sensitivity analysis

**Fig. 3:**
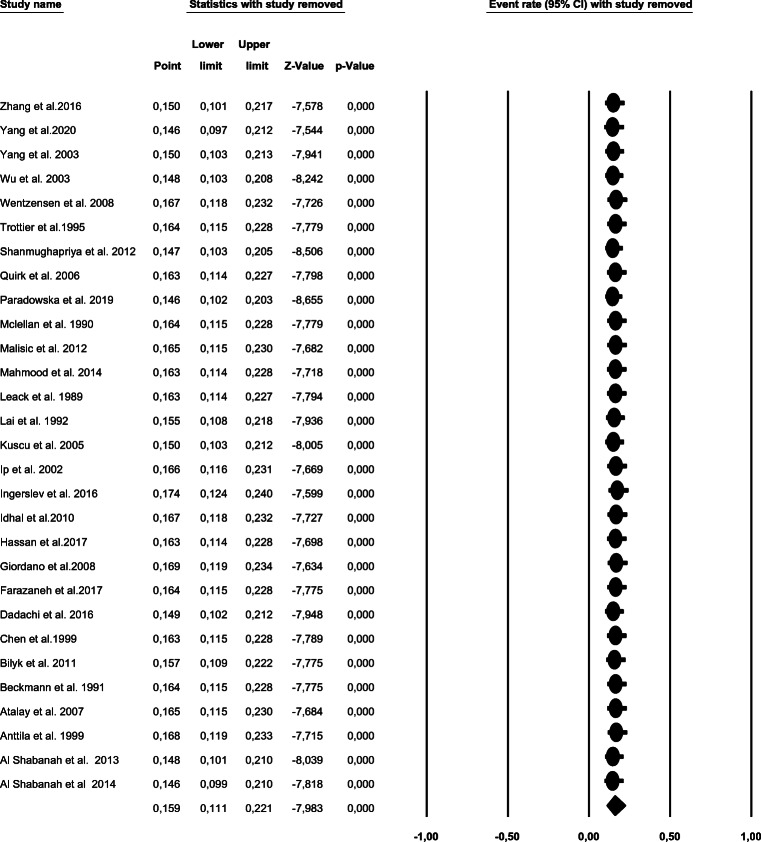
Sensitivity analysis showing the impact of exclusion of any study on the summary effect

To detect a potential bias related to the quality of the included studies, we performed a sensitivity analysis by calculating HPV prevalence when excluding one study at a time. Figure 3 shows that after the exclusion of each study, no significant effect on the overall prevalence of HPV in ovarian cancer patients was observed.

### Publication bias

**Fig. 4:**
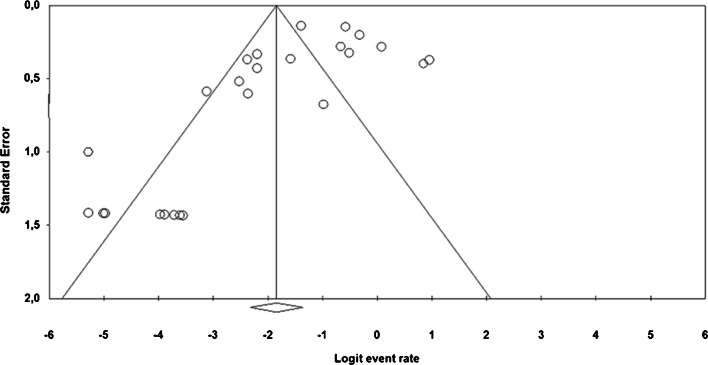
Funnel plot of studies included in the meta-analysis. The distribution of the studies (dots) is asymmetric suggesting an evidence of publication bias that is also found by Egger’s regression test (*P*=0.006) and Begg and Mazumdar rank correlation (*P*=0.5)

A visual inspection of the funnel plot showed an asymmetry, and a one-tailed p-value of Begg´s test and Egger´s regression test were 0.006 and 0.5, respectively (Figure 4). Suggesting that there is a significant publication bias.

## Discussion

Since the first report on HPV infection in ovarian cancer published by Kaufman et al. in 1987 [38] using southern blot hybridization, researchers have explored the potential involvement of HPV in ovarian cancerogenesis in several geographical regions and by more sensitive and specific methods. However, the association between HPV and ovarian cancer remains controversial. In the current meta-analysis, 2280 cases with ovarian cancer were investigated. In 19/29 studies where HPV was detected, 2 reported a cause-effect relation by detecting E6/E7 oncogenes expression or HR-HPV viral integration [21, 31]; while in 10 studies no HPV was found. The overall pooled prevalence of HPV was 15.9% (95% CI, 11–22), and the prevalence of detection ranged from 0 to 81%. This result is in accordance with previous meta-analyses published in 2013 [2, 10]. Nevertheless, in the present meta-analysis, six publications from the Middle East and four recent studies were additionally included.

This overall low prevalence of HPV found in ovarian cancer compared to the strongly HPV-associated malignancies, i.e., cervical cancer, might be explained by the adaptation and affinity of HPV to certain squamous cells in the cervical epithelium, or/and by the vulnerability of the transformation zone of the cervix. The latter is a niche of cells with an embryonic characteristic such as cuboidal epithelial cells, reserve cells, or potentially embryonal stem cells, which have been proposed as targeted cells by HPV infection [39–43]. However, HPV target cells in the ovarian epithelium have not been described yet.

Substantial heterogeneity between studies was found (*I*^2^=88%, *P* ≤0.001) and an asymmetric funnel plot was observed. These findings suggest a wide variation between study results. This is supported by major differences in terms of sample size, geographic distribution, type of specimen, detection methods, and the period of the study. Thus, to balance the selection bias, subgroup analyses were carried out.

It has been reported that geographical differences in the prevalence of HPV might be due to biological and geographic interaction between HPV variants and the host immunogenetic factors such as GST (glutathione-S-transferase), HLA (human leukocyte antigen), MDM2 (Mouse double minute 2 homolog), FAS (fatty acid synthase) gene promoter-670 and p53 codon 72 polymorphisms [44, 45]. Furthermore, impairment of the cellular immunity through malnutrition, inflammation, HIV, and microbial infection, genetic predisposition, lifestyles; might contribute to a higher incidence of HPV acquisition in some regions [46]. Moreover, in cervical cancer, an association between the prevalence of high risk-HPV infection in the general population and the incidence of this malignancy has been demonstrated [47]. Also, several researchers have found regional differences in the prevalence of HPV genotypes in invasive cervical cancer specimens. In a meta-analysis conducted on 85 studies, HPV16 was predominant in cervical squamous cell carcinoma, with a prevalence of 63% in North America and 46% in Asia [47]. Similarly, in a study conducted on 10,575 cases of invasive cervical cancer from 38 different countries tested for HPV, researchers found that HPV16 and 18 had the highest relative contributions in North America (79%) and Oceania (79%), followed by Africa (71%) and Asia (71%) [48]. In our subgroup analysis, stratified by geographical distribution, studies carried out in Asia (30.9%; 95% CI, 20–44) and Eastern Europe (29.3%; 95% CI, 4.4–78) had the highest prevalence of HPV in ovarian cancer detected while no HPV association was detected in North America. Furthermore, the meta-regression showed that geographic distribution explains the variation in worldwide HPV prevalence described in ovarian cancer.

FFPE blocks are a valuable source to conduct retrospective studies. However, a prolonged fixation time with formalin causes a cross-linking of proteins and nucleic acids and also random breakages in nucleotide sequences [49]. Besides, fixation protocols including non-buffered formalin may contribute to decreasing DNA integrity and reduced HPV detection rate. Furthermore, false positivity due to carryover contamination might occur if FFPE blocks were not processed under strict conditions [48]. On the other hand, frozen tissues allow better DNA preservation, but require constant low-temperature maintenance [50]. In the present study, the HPV detection rate was slightly higher when frozen tissues were used. Nevertheless, the subgroup and the meta-regression analyses showed that specimen type does not explain the variation of HPV prevalence.

Currently, HPV detection relies on molecular techniques that allow a high sensitivity and specificity of detection [51]. Usually, HPV detection by PCR can be performed either by using a single PCR primer set such as (i) L1 consensus primers MY09/MY1, GP5+/GP6+, or SPF10 primers followed by type-specific probe-based detection, direct sequencing or restriction-fragment length polymorphism (RFLP), (ii) by nested PCR using a combination of MY/GP primers; (iii) or by using type-specific primers [52]. Nevertheless, a single PCR primer set underestimates the prevalence of HPV positivity, but the combination of MY/GP in nested PCR is considered the most sensitive DNA-based test that allows detection of low levels of HPV [53]. In the present meta-analysis, PCR was the most often used technique (in 26/29 studies). However, the prevalence of HPV in ovarian tissues was the highest when 2 different techniques were combined (PCR and ISH or IHC) (31%; 95%CI, 24.4–38.5), and when nested PCR GP5+/GP6+ followed by reamplification with MY09/MY11 was used (29.1%; 95%CI, 25–33). Furthermore, a lower prevalence of HPV was found by using type-specific primers either by targeting E6/E7 regions or by using designed type-specific primers (12.7%; 95%CI, 4.7–10.3).

The overexpression of E6 and E7 oncoproteins are required to maintain malignant transformation in HPV-related cancers, which should be detected by specific primers for E6/E7 regions [54, 55]. Contrary to the well-established pathway of E6 and E7 in the cervical cancerogenesis, it has been discussed that the molecular mechanism of cancerogenesis in HPV-infected ovarian epithelium operates differently. This hypothesis was stated by Kisseljova et al. [53], who were unable to detect HPV16 DNA by E6/E7 primers despite finding HPV-association in their samples investigated. Also, Roos et al. [56] reported evidence of HPV presence in ovarian cancer in North America by detecting transcripts of HPV18 oncogenes in ovarian cancer transcriptomes (by RNA-Seq) from The Cancer Genome Atlas (TCGA) database. Hence, a more sensitive assay, based on mRNA HPV oncogene expression is needed, to clarify the role of this microorganism in ovarian cancerogenesis by comparing viral oncogene expression in cancer samples and adjacent normal tissues.

According to Bosch et al. [57], HPV16 is the most frequent genotypes detected in cervical cancer cases in most countries (50% to 60%), followed by HPV18 (10–20%), HPV45 (4–8%), and HPV31 (1–5%). Comparable results were found in our subgroup analysis stratified by HPV genotypes.

Coinfection with more than one HPV anogenital type was observed in more than 50% of sexually active women through their life [58]. Several studies showed that the presence of multiple HPV genotypes is associated with an increased risk of high-grade lesions at the cervix [59–61]. Besides, coinfections with alpha-9 genotypes increase the risk of cervical cancer 5.3 fold compared to a coinfection with alpha-7 genotypes [62]. Moreover, Senapati et al. [63], revealed that women infected with different genotypes in a phylogenetically related clad had a higher risk of cervical carcinoma in comparison to women infected with unrelated phylogenetic clad. Also, an in vitro study reported that coinfection of a single cell with HPV16 and HPV18 induces a replication interference between them [64]. Our meta-analysis revealed that an HPV16/18 coinfection was the most observed (17.2%) followed by HPV16/45 (9.6%). However, in one study where a coinfection with HPV16/18 was reported [29], 2 of 3 positive cases had an HPV16/18 coinfection that could potentially be a result of cross-contamination while sectioning FFPE blocks, knowing that no blank FFPE block was sectioned between cases to reveal any carryover contamination. Indeed, in order to prove an ethiological involvement of HPV in a certain cancer development, e.g. in HNSCC, recently a very elaborate and strict methodological process has been described involving (i) control for carryover by HPV free tissue sections between experimental blocks, (ii) measurement of HPV oncogene expression by detection of E6 and/or E7 mRNA, and (iii) detection p16^ink4a^ upregulation [65].

There were several limitations in our meta-analysis. First, in 22 studies, no control group was selected and the sample size was small in other studies, which does not allow to distinguish the difference between malignant and control groups. Second, few studies, reported HPV genotypes by histological type. The latter points out the need for more studies that take into account the histologic type of the malignancy. Third, no HPV oncogene expression was measured to investigate its role in ovarian cancerogenesis by comparing viral oncogene expression in malignant and normal tissues. Fourth, no carryover contamination control was performed while processing FFPE blocks, which might lead to an overestimation of HPV prevalence. Fifth, coinfection with more than one HPV genotype was evaluated in just 3 studies which did not allow us to investigate if the presence of multiple HPV genotypes could be associated with an increased risk of ovarian cancer. Sixth, the heterogeneity between studies was substantial and publication bias was found, this might be explained by methodological differences and the fact that scientific journals are more likely to publish studies that report positive results. Seventh, the majority of the included studies reported the studied population age in mean, so we were unable to perform a subgroup analysis by age group or perform meta-regression analysis, thus the discrepancy in prevalence could be due to differences in the age range of included studies. Furthermore, the data discrepancy might be also explained by the variation of HPV genotypes across geographical regions. Eighth, none of the included studies has mentioned if patients received HPV vaccine, which could be of interest to assess the efficacy of prophylactic vaccines for the potential prevention of HPV infections in the upper female genital tract.

Considering that HPV plays a role in a rising number of head and neck squamous cell carcinomas, it has been reported that patients with HPV+ tumors have a favorable prognosis, and HPV-selective therapies are under investigation. If there is a potential implication of HPV in ovarian cancerogenesis, treatment and maybe prognosis, HPV-association of ovarian cancer needs further investigation.

## Conclusion

Our meta-analysis suggests a great difference in the prevalence of HPV detected in ovarian cancer which is not seen in strongly HPV-associated cancers such as cervical cancer. However, further studies are needed, using more precise assays that identify active infection by testing HPV oncoprotein expression and informative biomarkers like p16 upregulation to prove the causality of HPV detection with cellular transformation. Also, other covariates such as ethnicity, age, and lifestyle have to be considered.

## Supplementary information

Supplementary Information 1 Characteristics of studies on HPV in ovarian cancer and borderline ovarian tumors included in the meta-analysis. (DOCX 25 kb)

## References

[1] Hassan ZK, Hafez MM, Kamel MM, Zekri ARN (2017). Human papillomavirus genotypes and methylation of CADM1, PAX1, MAL and ADCYAP1 genes in epithelial ovarian cancer patients. Asian Pac J Cancer Prev.

[2] Rosa MI, Silva GD, de Azedo Simões PW, Souza MV, Panatto AP, Simon CS, Madeira K, Medeiros LR (2013). The prevalence of human papillomavirus in ovarian cancer: a systematic review. Int J Gynecol Cancer.

[3] Cass I, Karlan BY (2010). Ovarian cancer symptoms speak out--but what are they really saying?. J Natl Cancer Inst.

[4] Reid BM, Permuth JB, Sellers TA (2017). Epidemiology of ovarian cancer: a review. Cancer Biol Med.

[5] Maringe C, Walters S, Butler J, Coleman MP, Hacker N, Hanna L, Mosgaard BJ, Nordin A, Rosen B, Engholm G, Gjerstorff ML, Hatcher J, Johannesen TB, McGahan CE, Meechan D, Middleton R, Tracey E, Turner D, Richards MA, Rachet B (2012). Stage at diagnosis and ovarian cancer survival: evidence from the International Cancer Benchmarking Partnership. Gynecol Oncol.

[6] Ingerslev K, Hogdall E, Skovrider-Ruminski W, Schnack TH, Karlsen MA, Nedergaard L, Hogdall C, Blaakær J (2016). High-risk HPV is not associated with epithelial ovarian cancer in a Caucasian population. Infect Agent Cancer.

[7] Yang HJLV, Tsang PC, Yip AM, Ng TY, Cheung AN (2003). Comparison of human papillomavirus DNA levels in gynecological cancers: implication for cancer development. Tumour biology the journal of the International Society for Oncodevelopmental Biology and Medicine.

[8] Farzaneh F, Nadji SA, Khosravi D, Hosseini MS, Hashemi Bahremani M, Chehrazi M, Bagheri G, Sigaroodi A, Haghighatian Z (2017). Lack of HPV in benign and malignant epithelial ovarian tumors in Iran. Asian Pac J Cancer Prev.

[9] Anttila M, Syrjänen S, Ji H, Saarikoski S, Syrjänen K (1999). Failure to demonstrate human papillomavirus DNA in epithelial ovarian cancer by general primer PCR. Gynecol Oncol.

[10] Svahn MF, Faber MT, Christensen J, Norrild B, Kjaer SK (2014). Prevalence of human papillomavirus in epithelial ovarian cancer tissue. A meta-analysis of observational studies. Acta Obstet Gynecol Scand.

[11] Higgins JP, Thompson SG (2002). Quantifying heterogeneity in a meta-analysis. Stat Med.

[12] Sutton AJ, Abrams KR, Jones DR (2001). An illustrated guide to the methods of meta-analysis. J Eval Clin Pract.

[13] Higgins JP, Thompson SG, Deeks JJ, Altman DG (2003). Measuring inconsistency in meta-analyses. Bmj.

[14] Quirk JT, Kupinski JM, DiCioccio RA (2006). Analysis of ovarian tumors for the presence of human papillomavirus DNA. J Obstet Gynaecol Res.

[15] Chen TR, Chan PJ, Seraj IM, King A (1999). Absence of human papillomavirus E6-E7 transforming genes from HPV 16 and 18 in malignant ovarian carcinoma. Gynecol Oncol.

[16] Trottier AM, Provencher D, Mes-Masson AM, Vauclair R, Coutlée F (1995). Absence of human papillomavirus sequences in ovarian pathologies. J Clin Microbiol.

[17] Beckmann AM, Sherman KJ, Saran L, Weiss NS (1991). Genital-type human papillomavirus infection is not associated with surface epithelial ovarian carcinoma. Gynecol Oncol.

[18] McLellan R, Buscema J, Guerrero E, Shah KV, Woodruff JD, Currie JL (1990). Investigation of ovarian neoplasia of low malignant potential for human papillomavirus. Gynecol Oncol.

[19] Leake JF, Woodruff JD, Searle C, Daniel R, Shah KV, Currie JL (1989). Human papillomavirus and epithelial ovarian neoplasia. Gynecol Oncol.

[20] Atalay F, Taskiran C, Taner MZ, Pak I, Or M, Tuncer S (2007). Detection of human papillomavirus DNA and genotyping in patients with epithelial ovarian carcinoma. J Obstet Gynaecol Res.

[21] Al-Shabanah OA, Hafez MM, Hassan ZK, Sayed-Ahmed MM, Abozeed WN, Al-Rejaie SS, Alsheikh AA (2013). Human papillomavirus genotyping and integration in ovarian cancer Saudi patients. Virol J.

[22] Al-Shabanah OA, Hafez MM, Hassan ZK, Sayed-Ahmed MM, Abozeed WN, Alsheikh A, Al-Rejaie SS (2014). Methylation of SFRPs and APC genes in ovarian cancer infected with high risk human papillomavirus. Asian Pac J Cancer Prev.

[23] Mahmood FM, Kadhim HS, Mousa Al Khuzaee LR (2014). Detection of human papillomavirus-16 e6-oncoprotein in epithelial ovarian tumors samples of iraqi patients. Jundishapur J Microbiol.

[24] Kuscu E, Ozdemir BH, Erkanli S, Haberal A (2005). HPV and p53 expression in epithelial ovarian carcinoma. Eur J Gynaecol Oncol.

[25] Dadashi M, Eslami G, Faghihloo E, Pourmohammad A, Hosseini J, Taheripanah R, Arab-Mazar Z (2017). Detection of human papilloma virus type 16 in epithelial ovarian tumors samples. Arch Clin Infect Dis.

[26] Zhang P-P, Zhou L, Cao J-S, Li Y-P, Zeng Z, Sun N, Shen L, Zhu H-Y, Ruan Y, Zha W-T, Wang X-Y, Zhang K-Q, Zhang R (2016). Possible epithelial ovarian cancer association with HPV18 or HPV33 infection. Asian Pac J Cancer Prev.

[27] Wu QJ, Guo M, Lu ZM, Li T, Qiao HZ, Ke Y (2003). Detection of human papillomavirus-16 in ovarian malignancy. Br J Cancer.

[28] Ip SM, Wong LC, Xu CM, Cheung AN, Tsang PC, Ngan HY (2002). Detection of human papillomavirus DNA in malignant lesions from Chinese women with carcinomas of the upper genital tract. Gynecol Oncol.

[29] Lai CH, Hsueh S, Lin CY, Huang MY, You GB, Chang HC, Pao CC (1992). Human papillomavirus in benign and malignant ovarian and endometrial tissues. Int J Gynecol Pathol.

[30] Yang X, You Q, Yao G, Geng J, Ma R, Meng H (2020). Evaluation of p16 in epithelial ovarian cancer for a 10-year study in Northeast China: significance of HPV in correlation with PD-L1 expression. Cancer Manag Res.

[31] Shanmughapriya S, Senthilkumar G, Vinodhini K, Das BC, Vasanthi N, Natarajaseenivasan K (2012). Viral and bacterial aetiologies of epithelial ovarian cancer. Eur J Clin Microbiol Infect Dis.

[32] Malisic E, Jankovic R, Jakovljevic K (2012). Detection and genotyping of human papillomaviruses and their role in the development of ovarian carcinomas. Arch Gynecol Obstet.

[33] Bilyk OO, Pande NT, Buchynska LG (2011). Analysis of p53, p16(INK4a), pRb and Cyclin D1 expression and human papillomavirus in primary ovarian serous carcinomas. Exp Oncol.

[34] Idahl A, Lundin E, Elgh F, Jurstrand M, Møller JK, Marklund I, Lindgren P, Ottander U (2010). Chlamydia trachomatis, Mycoplasma genitalium, Neisseria gonorrhoeae, human papillomavirus, and polyomavirus are not detectable in human tissue with epithelial ovarian cancer, borderline tumor, or benign conditions. Am J Obstet Gynecol.

[35] Wentzensen N, du Bois A, Kommoss S, Pfisterer J, von Knebel DM, Schmidt D, Kommoss F (2008). No metastatic cervical adenocarcinomas in a series of p16INK4a-positive mucinous or endometrioid advanced ovarian carcinomas: an analysis of the AGO Ovarian Cancer Study Group. Int J Gynecol Pathol.

[36] Giordano G, D’Adda T, Gnetti L, Froio E, Merisio C, Melpignano M (2008). Role of human papillomavirus in the development of epithelial ovarian neoplasms in Italian women. J Obstet Gynaecol Res.

[37] Paradowska E, Jabłońska A, Studzińska M, Wilczyński M, Wilczyński JR (2019). Detection and genotyping of CMV and HPV in tumors and fallopian tubes from epithelial ovarian cancer patients. Sci Rep.

[38] Kaufman RH, Bornstein J, Gordon AN, Adam E, Kaplan AL, Adler-Storthz K (1987). Detection of human papillomavirus DNA in advanced epithelial ovarian carcinoma. Gynecol Oncol.

[39] Herfs M, Soong TR, Delvenne P, Crum CP (2017). Deciphering the multifactorial susceptibility of mucosal junction cells to HPV infection and related carcinogenesis. Viruses.

[40] Herfs M, Vargas SO, Yamamoto Y, Howitt BE, Nucci MR, Hornick JL, Mckeon FD, Xian W, Crum CP (2013). A novel blueprint for ‘top down’ differentiation defines the cervical squamocolumnar junction during development, reproductive life, and neoplasia. J Pathol.

[41] Herfs M, Yamamoto Y, Laury A, Wang X, Nucci MR, McLaughlin-Drubin ME, Münger K, Feldman S, McKeon FD, Xian W, Crum CP (2012). A discrete population of squamocolumnar junction cells implicated in the pathogenesis of cervical cancer. Proc Natl Acad Sci U S A.

[42] Organista-Nava J, Gómez-Gómez Y, Garibay-Cerdenares OL, Leyva-Vázquez MA, Illades-Aguiar B (2019). Cervical cancer stem cell-associated genes: prognostic implications in cervical cancer (Review). Oncol Lett.

[43] Reich O, Regauer S (2015). Two major pathways of recurrent high-grade squamous intraepithelial lesions of the cervix. Int J Cancer.

[44] Clifford GM, Gallus S, Herrero R, Muñoz N, Snijders PJF, Vaccarella S, Anh PTH, Ferreccio C, Hieu NT, Matos E, Molano M, Rajkumar R, Ronco G, de Sanjosé S, Shin HR, Sukvirach S, Thomas JO, Tunsakul S, Meijer C, Franceschi S (2005). Worldwide distribution of human papillomavirus types in cytologically normal women in the International Agency for Research on Cancer HPV prevalence surveys: a pooled analysis. Lancet.

[45] Nunobiki O, Ueda M, Toji E, Yamamoto M, Akashi K, Sato N, Izuma S, Torii K, Tanaka I, Okamoto Y, Noda S (2011). Genetic polymorphism of cancer susceptibility genes and HPV infection in cervical carcinogenesis. Pathol Res Int.

[46] Liu G, Sharma M, Tan N, Barnabas RV (2018). HIV-positive women have higher risk of human papilloma virus infection, precancerous lesions, and cervical cancer. AIDS.

[47] Bruni L, Diaz M, Castellsagué M, Ferrer E, Bosch FX, de Sanjosé S (2010). Cervical human papillomavirus prevalence in 5 continents: meta-analysis of 1 million women with normal cytological findings. J Infect Dis.

[48] de Sanjose S, Quint WG, Alemany L, Geraets DT, Klaustermeier JE, Lloveras B, Tous S, Felix A, Bravo LE, Shin HR, Vallejos CS, de Ruiz PA, Lima MA, Guimera N, Clavero O, Alejo M, Llombart-Bosch A, Cheng-Yang C, Tatti SA, Kasamatsu E, Iljazovic E, Odida M, Prado R, Seoud M, Grce M, Usubutun A, Jain A, Suarez GA, Lombardi LE, Banjo A, Menéndez C, Domingo EJ, Velasco J, Nessa A, Chichareon SC, Qiao YL, Lerma E, Garland SM, Sasagawa T, Ferrera A, Hammouda D, Mariani L, Pelayo A, Steiner I, Oliva E, Meijer CJ, Al-Jassar WF, Cruz E, Wright TC, Puras A, Llave CL, Tzardi M, Agorastos T, Garcia-Barriola V, Clavel C, Ordi J, Andújar M, Castellsagué X, Sánchez GI, Nowakowski AM, Bornstein J, Muñoz N, Bosch FX (2010). Human papillomavirus genotype attribution in invasive cervical cancer: a retrospective cross-sectional worldwide study. Lancet Oncol.

[49] Nagahashi M, Shimada Y, Ichikawa H, Nakagawa S, Sato N, Kaneko K, Homma K, Kawasaki T, Kodama K, Lyle S, Takabe K, Wakai T (2017). Formalin-fixed paraffin-embedded sample conditions for deep next generation sequencing. J Surg Res.

[50] Gao XH, Li J, Gong HF, Yu GY, Liu P, Hao LQ, Liu LJ, Bai CG, Zhang W (2020) Comparison of fresh frozen tissue with formalin-fixed paraffin-embedded tissue for mutation analysis using a multi-gene panel in patients with colorectal cancer. Front Oncol 10(310). 10.3389/fonc.2020.00310 10.3389/fonc.2020.00310 PMC7083147 32232001

[51] Erhart SMM, Rivero ERC, Bazzo ML, Onofre ASC (2016). Comparative evaluation of the GP5+/6+, MY09/11 and PGMY09/11 primer sets for HPV detection by PCR in oral squamous cell carcinomas. Exp Mol Pathol.

[52] Eide ML, Debaque H (2012). HPV detection methods and genotyping techniques in screening for cervical cancer. Ann Pathol.

[53] Kisseljova N, Zhordania K, Fedorova M, Katargin A, Valeeva A, Pajanidi J, Pavlova L, Khvan O, Vinokurova S (2019). Detection of human papillomavirus prevalence in ovarian cancer by different test systems. Intervirology.

[54] von Knebel DM, Rittmüller C, Hausen HZ, Dürst M (1992). Inhibition of tumorigenicity of cervical cancer cells in nude mice by HPV e6-e7 anti-sense RNA. Int J Cancer.

[55] Doorbar J (2006). Molecular biology of human papillomavirus infection and cervical cancer. Clin Sci.

[56] Roos P, Orlando PA, Fagerstrom RM, Pepper JW (2015). In North America, some ovarian cancers express the oncogenes of preventable human papillomavirus HPV-18. Sci Rep.

[57] Bosch FX, de Sanjosé S (2007). The epidemiology of human papillomavirus infection and cervical cancer. Dis Markers.

[58] Carrillo-García A, Ponce-de-León-Rosales S, Cantú-de-León D, Fragoso-Ontiveros V, Martínez-Ramírez I, Orozco-Colín A, Mohar A, Lizano M (2014). Impact of human papillomavirus coinfections on the risk of high-grade squamous intraepithelial lesion and cervical cancer. Gynecol Oncol.

[59] Dickson EL, Vogel RI, Bliss RL, Downs LS (2013). Multiple-type human papillomavirus (HPV) infections: a cross-sectional analysis of the prevalence of specific types in 309,000 women referred for HPV testing at the time of cervical cytology. Int J Gynecol Cancer.

[60] Tran LT-H, Tran LT, Bui TC, Le DT-K, Nyitray AG, Markham CM, Swartz MD, Vu-Tran CB, Hwang L-Y (2015). Risk factors for high-risk and multi-type human papillomavirus infections among women in Ho Chi Minh City, Vietnam: a cross-sectional study. BMC Womens Health.

[61] Figueiredo Alves RR, Turchi MD, Santos LE, EMdB G, MMD G, MSC S, Villa LL, Costa MC, MAR M, MdFdC A (2013). Prevalence, genotype profile and risk factors for multiple human papillomavirus cervical infection in unimmunized female adolescents in Goiânia, Brazil: a community-based study. BMC Public Health.

[62] Resende LS, Rabelo-Santos SH, Sarian LO, Figueiredo Alves RR, Ribeiro AA, Zeferino LC, Derchain S (2014). A portrait of single and multiple HPV type infections in Brazilian women of different age strata with squamous or glandular cervical lesions. BMC Infect Dis.

[63] Senapati R, Nayak B, Kar SK, Dwibedi B (2017). HPV genotypes co-infections associated with cervical carcinoma: special focus on phylogenetically related and non-vaccine targeted genotypes. PLoS One.

[64] Mori S, Kusumoto-Matsuo R, Ishii Y, Takeuchi T, Kukimoto I (2014). Replication interference between human papillomavirus types 16 and 18 mediated by heterologous E1 helicases. Virol J.

[65] Baboci L, Holzinger D, Boscolo-Rizzo P, Tirelli G, Spinato R, Lupato V, Fuson R, Schmitt M, Michel A, Halec G, Da Mosto MC, Pawlita M, Del Mistro A (2016). Low prevalence of HPV-driven head and neck squamous cell carcinoma in North-East Italy. Papillomavirus Res.

