# Supplementary material for: Prevalence of human papillomavirus detection in ovarian cancer: a meta-analysis
Source: Eur J Clin Microbiol Infect Dis. 2021 Jun 4;40(9):1791–802. doi: 10.1007/s10096-021-04282-7 (PMC8346400; doi:10.1007/s10096-021-04282-7)
Supplement: Supplementary file 1 — Characteristics of studies on HPV in ovarian cancer and borderline ovarian tumors included in the meta-analysis. (DOCX 25 kb) [file 10096_2021_4282_MOESM1_ESM.docx]

**Prevalence of Human Papillomavirus detection in Ovarian Cancer: A Meta-Analysis**

Soumia Cherif ¹ ², Abdessamad Amine^2^, Sarah Thies¹, Eliane T. Taube^3^, Elena Ioana Braicu¹, Jalid Sehouli^1^, Andreas M. Kaufmann^1*^

¹ Charité – Universitätsmedizin Berlin, corporate member of Freie Universität Berlin and Humboldt-Universität zu Berlin, Department of Gynecology, Augustenburger Platz 1, 13353 Berlin, Germany

² Laboratory of Biochemistry, Environment, and Agrifood, Faculty of Sciences and Techniques-Mohammedia, Hassan II University, Casablanca, Morocco

^3^ Charité – Universitätsmedizin Berlin, corporate member of Freie Universität Berlin and Humboldt-Universität zu Berlin, Institute for Pathology, Charitéplatz 1, 10117 Berlin, Germany

**Supplementary information 1:** **Characteristics of studies on HPV in ovarian cancer and borderline ovarian tumors included in the meta-analysis.**

| **Reference**  **(Year)** | **Country** | **Method of Detection** | **Specimen** | **Histological Types** | **HPV+/Total cases** | **HPV Detected** |
| --- | --- | --- | --- | --- | --- | --- |
| Yang et al. ^(30)^ (2020) | China | PCR (L1 Consensus primers GP5+/GP6+) | FFPE | 208 Serous adenocarcinoma  48 Mucinous adenocarcinoma  54 Others | 78/310 | ND |
| Paradowska et al. ^(37)^(2019) | Poland | qRT-PCR(HR-HPV E6 oncoprotein)  High risk Human Papillomavirus Multiplex screening genesig kit | Frozen tissues | 27 Epithelial ovarian cancers:  20 High grade serous  7 Other types  2Bordeline  2 Mucinous adenocarcinoma  2 Clarocellular adenocarcinoma  1 Undifferentiated carcinoma | 22/27 | 19/27 HPV16  2/27 HPV6  1/27 HPV45 |
| Farazaneh et al.^(8)^  (2017) | Iran | PCR (L1 Consensus primers MY09/MY11; GP5+/GP6+) | FFPE | 26 Epithelial ovarian cancers | 0/26 | 0 |
| Hassan et al.^(1)^  (2017) | Egypt | PCR (L1 Consensus primers MY09/MY11; GP5+/GP6+ ) | FFPE | 100 Epithelial ovarian cancer tissues | 10\100 | 5/10 HPV 16  4/10 HPV 18  1/10 HPV 33 |
| Ingerslev et al. ^(6)^ | Denmark | Q-PCR (specific primers for E6/E7 region of the HPV16/18) | FFPE | 198 Epithelial ovarian cancer tissues:  163 Serous  15 Endometroid  11 Mucinous  9 Clear cell | 1/198 | 1/1 HPV18 |
| Dadachi et al. ^(25)^  (2017) | Iran | PCR (L1 Consensus primers GP5+/GP6+)followed by sequencing | FFPE | 70 Epithelial ovarian cancer tissues | 25/70 | 25/70HPV16 |
| Zhang et al. ^(26)^  (2016) | China | PCR: i) L1 consensus primers GP5+/GP6+  ii) specific primers for HPV18 and 33 | FFPE | 322 Epithelial ovarian cancer tissues | 64/322 | 25/64 HPV18  39/64 HPV33 |
| Al Shabanah et al.^(22)^ (2014) | Saudi Arabia | PCR (L1 Consensus primers MY09/MY11; GP5+/GP6+) | FFPE | unknown | 72/200 | 23/72 HPV18 20/72 HPV16  3/72 HPV45  11/72 HPV16/18  10/72 HPV16/45  2/72 HPV18/45 3/32HPV16/18/45 |
| Mahmood et al. ^(23)^  (2014) | Iraq | IHC (HPV 16-E6-oncoprotein) | FFPE | 4 Bordeline  2 Serous  1 Mucinous  1 endometroid  31 Ovarian cancer :  26 Serous  3 Endometroid  2 Mucinous | 3/31  0/4 | 3/3 HPV16 |
| Al Shabanah et al. ^(21)^ (2013) | Saudi Arabia | PCR (L1 Consensus primers MY09/MY11; GP5+/GP6+) | FFPE | unknown | 42/100 | 18/42 HPV16 11/42 HPV18 7/42 HPV16/18  3/42 HPV45  1/42 HPV16/45  1/42 HPV18/45 1/42HPV16/18/45 |
| Malisic et al. ^(32)^  (2012) | Serbia | PCR (L1 primers GP5+/GP6+) followed by sequencing | Frozen tissues | 54 Ovarian cancer tissues:  35 Serous  7 Endometroid  4 Clear cell  3Adenocarcinoma Nos  2 Mucinous  2Undifferentiated  1 Mixed | 4/54 | 4/4 HPV16 |
| Shanmughapriya et al. ^(31)^ (2012) | India | PCR:  i) L1 Consensus primers MY09/MY11;  ii) type-specific primers for HPV16, 18, 52, 6, and 11;  iii) consensus primers PU-1 M/PU-2R (specific for HR-HPV31, 33, 52b and 58), PU-31B/PU-2R (specific for other LR-HPV),  iv)consensus primers for E6 /E7 proteins, and E2 proteins | frozen tissues | 6 Bordeline  24 Epithelial ovarian cancer tissues:  12 Serous  6 Mucinous  6 Endometroid | 15/24  6/6 | 15/15 HPV 6  6/6 HPV 6 |
| Bilyk et al. ^(33)^  (2011) | Ukrain | IHC (HPV16/ 18 E6)  PCR (specific primers for HPV16; HPV18) | FFPE | 53 Serous ovarian cancer | 9\53 | 4/9 HPV 16  5/9 HPV 18 |
| Idhal et al. ^(31)^  (2010) | Sweden | PCR (L1 Consensus GP5+/GP6+) | Frozen tissues | 20 Bordeline  10 Serous  10 Mucinous  52 ovarian cancer tissues :  33 Serous  7 Endometroid  5 Mucinous  3 Clear cell  2 Mixed  2 Undifferenciated | 0/72 | 0 |
| Wentzensen et al. ^(35)^ (2008) | Germany | PCR:  i)L1 Consensus primers MY09/MY11; GP5+/GP6+)  ii) HPV16\18 specific primers | Frozen Tissues | 74 Epithelial ovarian cancer:  24 Mucinous  50 Endometroid | 0/74 | 0 |
| Giordano et al. ^(36)^  (2008) | Italy | PCR (L1 consensus primers GP5+/GP6+) | FFPE | 21 Bordeline  13 Serous  8 Mucinous  50 Epithelial ovarian cancers  25 Serous  25 Mucinous | 2/21  1/50 | ND |
| Atalay et al. ^(20)^  (2007) | Turkey | PCR (L1 Consensus primers MY09/MY11) | FFPE | 94 epithelial ovarian cancers:  76 Serous  9 Mucinous  4Undifferentiated  3 Unclassified  1 Endometroid  1 Mixed | 8/94 | 6/8 HPV6  2/8 HPV 33 |
| Quirk et al. ^(14)^  (2006) | USA | PCR (E6 transforming region of HPV types 16, 18, and 33)  HPV detection sets (PanVera, Madison, WI, USA) | Frozen tissues | 17 Ovarian cancer tissues:  13 Serous  3 Endometroid  1 Carcinosarcoma | 0/17 | 0 |
| Kuscu et al. ^(24)^  (2005) | Turkey | ISH, IHC | FFPE | 40 Epithelial ovarian cancer tissues | 15\40 | UNK |
| Wu et al. ^(27)^  (2003) | China | ISH , IHC | FFPE | 50 Ovarian cancer tissues :  19 Mucinous  24 Serous  5 Endometrioid  2 Undifferentiated carcinomas | 26/50 | 26/26 HPV 16 |
| Yang et al. ^(7)^  (2003) | China | q-PCR (specific primers for HPV16/18) | Frozen tissues | 56 Ovarian cancer tissues :  21 Serous  14 Endometroid  11 Mucinous  6 Clear cell  4 Undifferenciated | 19/56 | 18/19HPV16  1/19 HPV18 |
| Ip et al. ^(28)^  (2002) | China | PCR (L1 Consensus primers MY09/MY11; GP5+/GP6+ ; specific primers for E6 for HPV16/18) | Fresh tissues | 9 Bordeline  7 Mucinous  2 serous  51 Epithelial ovarian cancer tissues:  15 Serous  11 Mucinous  7 Clear-cell  7 Endometrioid  6 Adenocarcinomas  5 Epithelial carcinomas | 2/9  2/51 | 1/2 HPV18  1/2 HPV16  2/2 HPV 16 |
| Anttila et al. ^(9)^  (1999) | Finland | PCR (general primers GP5+/GP6+) | FFPE | 98 epithelial ovarian cancers  31 Serous  28 Endometroiod  12 Clear cell  12 Mixed  8 Mucinous  6 Unclassified  1 Brenner | 0/98 | 0 |
| Chen et al. ^(15)^  (1999) | USA | PCR (specific primers for E6-E7 for HPV16/18) | FFPE | 20 Epithelial ovarian cancers | 0/20 | 0 |
| Trottier et al. ^(16)^  (1995) | Canada | PCR (L1 Consensus primers MY09/MY11) | Fresh tissues | 1 Bordeline  23 Ovarian cancer tissues:  15 Cystadenocarcinoma  3 Clear cell  3 Endometroid  1 Serous  1 Mucinous | 0/24 | 0 |
| Beckmann et al. ^(17)^  (1991) | USA | PCR (general primers MY09/MY11) | FFPE | 8 Bordeline  6 Serous  1 Endometroid  1 Mucinous  18 Epithelial ovarian cancer  9 Serous  3 Mucinous  2 Mixed  2 Clear cell  1 Brenner  1 Endometroid | 0/26 | 0 |
| Lai et al. ^(29)^  (1992) | China | PCR (specific primers for E6 for HPV16/18 ) | FFPE | 11 Epithelial ovarian cancer tissues:  7 Serous  3 Mucinous  1 Mixed | 3/11 | 2/3 HPV16/18  1/3 HPV18 |
| Mclellan et al. ^(18)^  (1990) | USA | PCR (E6 transforming region of HPV types 16,18,6 and HPV11) | FFPE | 24 Ovarian cancer tissues | 0/24 | 0 |
| Leack et al. ^(19)^  (1989) | USA | PCR (specific primers for HPV6/11)  Southern hybridization (specific primers for HPV 6,16,18,31,35) | Frozen Tissues | 18 Epithelial ovarian cancer tissues:  12 Adenocarcinomas  4 Serous  1 Mixed  1 Mucinous | 0/18 | 0 |

PCR: polymerase chain reaction; HPV: human papillomavirus; FFPE: Formalin-Fixed Paraffin-Embedded;

ISH: In-situ hybridization; ND: not determined; Nos : not otherwise specified.
